# Provision of smoking cessation support for pregnant women in England: results from an online survey of NHS stop smoking services for pregnant women

**DOI:** 10.1186/1472-6963-14-107

**Published:** 2014-03-04

**Authors:** Samantha J Fahy, Sue Cooper, Tim Coleman, Felix Naughton, Linda Bauld

**Affiliations:** 1 Division of Primary Care, U.K. Centre for Tobacco and Alcohol Studies and National Institute for Health Research School for Primary Care Research, University of Nottingham, Nottingham, England; 2 Behavioural Science Group, University of Cambridge, Cambridge, England; 3 Institute for Social Marketing and U.K. Centre for Tobacco and Alcohol Studies, University of Stirling, Stirling, Scotland

**Keywords:** Smoking cessation, Smoking in pregnancy, Service evaluation, Nicotine replacement therapy, Online survey

## Abstract

**Background:**

Smoking during pregnancy is a major public health concern and an NHS priority. In 2010, 26% of UK women smoked immediately before or during their pregnancy and 12% smoked continuously. Smoking cessation support is provided through free at the point of use Stop Smoking Services for Pregnant women (SSSP). However, to date, little is known of how these services provide support across England. The aim of this study was to describe the key elements of support provided through English SSSP.

**Methods:**

SSSP managers were invited to participate in this survey by email. Data were then collected via an online questionnaire; one survey was completed for each SSSP. Up to four reminder emails were sent over a two month period.

**Results:**

86% (121 of 141) of services completed the survey. Responding services were, on average, larger than non-responding services in terms of the number of pregnant women setting quit dates and successfully quitting (p < 0.01). In line with the 2010 NICE guidelines, Stop Smoking in Pregnancy and following Childbirth, one in five SSSP identified pregnant smokers using carbon monoxide (CO) testing and refer via an opt-out pathway. All services offered nicotine replacement therapy (NRT) to pregnant women and 87% of services also offered dual therapy NRT, i.e. combination of a patch and short acting NRT product.. The 2010 NICE guidelines note that services should be flexible and client-centred. Consistent with this, SSSP offer pregnant women a range of support types (median 4) including couple/family, group (open or closed) or one-to-one. These are available in a number of locations (median 5), including in community venues, clinics and women’s homes.

**Conclusions:**

English Stop Smoking Services offer behavioural support and pharmacotherapy to pregnant women motivated to quit smoking. Interventions provided are generally evidence-based and delivered in a variety of both social and health care settings.

## Background

Smoking during pregnancy is a major public health concern. Improving health outcomes of the next generation is particularly of current global importance during the economic downturn since health is key to sustainable economic development [1]. The prevalence of smoking during pregnancy varies between countries and reflects variations in tobacco use amongst women. In many, low and middle income countries, smoking in pregnancy is an emerging problem [2,3]. In developed countries, the problem is established with the prevalence of smoking during pregnancy reported at around 10% in Canada [4] and Japan [5], 14% in the USA [6], 30-35% in Spain [7] and in the UK in 2010, 12% of women smoked throughout their pregnancy [8]. As in many high income countries, smoking prevalence during pregnancy in the UK has decreased over the past decade [4,8,9]. However, data from Canada and Australia show the rate of decline is markedly less amongst women from lower socioeconomic groups, compared to those from more affluent groups [9,10].

The literature suggests that brief advice to stop smoking this has little impact on cessation outcomes for pregnant women [11,12]. More intensive psychosocial interventions, however, have been shown to be effective for smoking cessation during pregnancy and also in reducing adverse perinatal outcomes [3]. In the UK, such support is available to pregnant women via Stop Smoking Services which are free at the point of use under the National Health Service (NHS). Stop smoking services for all smokers were originally introduced in 1999 to provide support to all smokers who requested this. They were the first national smoking cessation services to be set up globally and have served as a model to those establishing similar programmes elsewhere [13], in line with the recommendations in Article 14 of the Framework Convention on Tobacco Control [14].

Within the UK’s cessation services, tailored provision for pregnant women (Stop Smoking Services for Pregnant women; SSSP) began to be developed from 2000. An observational audit of Scottish SSSP outcomes suggested that delivering support through services developed solely for pregnant women may be more effective than supporting pregnant women through generic services, which support all smokers [15]. In 2010, the first UK guidelines were released on how to stop smoking in pregnancy and following childbirth [16]. These guidelines make recommendations about, not only which cessation interventions are effective for pregnant smokers but also how health providers should try to identify and engage with pregnant women to offer these to them. One recommendation, for example, is that SSSP should systematically introduce into routine care, the use of exhaled carbon monoxide to identify pregnant smokers; the guidance also suggests that where CO levels indicate that women are smokers, as a default they should be offered NHS stop smoking support. To date, no research has been conducted to examine how specialist smoking in pregnancy services support quit attempts in England. We therefore conducted a national survey to assess how SSSP are configured, how they receive and accept referrals of pregnant smokers, and how they then deliver smoking cessation support to pregnant women in England.

## Methods

### Survey design

The survey was developed in consultation with specialists in smoking in pregnancy, SSSP managers and policy makers knowledgeable about SSSP. It was designed to be completed by each SSSP manager and covered six main areas: service configuration, identification and referral of pregnant smokers, methods of engaging with pregnant women, information about service users, budgets and costs and service delivery. The online version of the survey was designed and implemented using Qualtrics software (Qualtrics Labs Inc., Provo, UT).

The online survey was piloted with 14 SSSP managers. Only a minor alteration was made to the survey as a result of this; therefore pilot responses were analysed together with those from the final survey. All questions in the survey related to the 12 month reporting period from April 2010 to March 2011. A link to the online survey was sent to all SSSP managers via email; each respondent received a unique username to access and complete it. Non-responders were sent up to four reminder emails over a three month period and if there was still no response, respondents were contacted by telephone and offered alternative means of completing the survey (via hard copy, electronic copy or by telephone). A ‘Thank you’ email was automatically sent after respondents had submitted their online survey responses. Nine SSSP were successfully followed up by telephone in order to address problems with missing data.

### Survey dissemination and completion

All managers and commissioners of the 141 NHS SSSP services that were identified across England including the Isle of Wight and the Isle of Man were contacted by email to inform them about the survey and to obtain agreement to complete the survey. The introduction of a ‘commissioner-provider’ system in the English NHS has brought about the introduction of ‘payments by results’ (PbR) services in which payment to Stop Smoking Services are based on the number of successful quitters [17,18]. In our survey, NHS standard contract Stop Smoking Services that had become PbR services, principally located in the West Midlands of England, [19] were included but the approximately 60 private PbR providers [19] were not.

This work was conducted according to the tenets of the Declaration of Helsinki. The results presented were obtained as part of a service evaluation therefore ethics approval was not required.

### Data handling and analysis

All data were cleaned and analysed using Stata 11 (StataCorp LP, College Station, TX). Responses are presented as descriptive statistics in the order in which the questions appeared in the online survey. Response rate to the budgets and costs section was low; these data will not be presented.

### Non-survey data

SSSP are required to report publicly-available service delivery data quarterly to the English Department of Health; this is then made [20]. Responding services were compared with non-responding services using the following routine data: service performance data, specifically, number of pregnant women setting a quit date and number of pregnant women remaining quit at four weeks [20], index of multiple deprivation (IMD) [21] and smoking at time of delivery (SaToD; available at http://www.hscic.gov.uk/searchcatalogue?productid=13675&q=title%3a%22Statistics+on+Women%27s+Smoking+Status+at+Time+of+Delivery%22&sort=Relevance&size=10&page=1#top). IMD provides a deprivation score for each small area in England by using economic, social and housing indicators to identify the degree of poverty; higher IMD scores indicate higher levels of deprivation [21]. Self-reported and carbon monoxide (CO) validated quit rates were estimated from service performance data [20] as the proportion of pregnant women setting a quit date who remained quit at four weeks. For non-normally distributed variables, Wilcoxon-Mann–Whitney tests were used to assess potential differences in the underlying distribution between the two groups. For normally distributed variables, two sample t-tests were used to assess potential differences in the means between the two groups.

## Results

One hundred and forty one NHS SSSP were identified across England and the Isles of Wight and Man. Of these, 134 (95%) provided contact details of a representative to complete the survey (usually the service manager or pregnancy lead). Responses were received from a total of 121 SSSP (104 online, 9 telephone and 8 by hard copy or email), representing an 86% response rate. These services provide support to smokers in all areas of the country; areas covered by SSSP were approximately coterminous with those covered by the 152 Primary Care Trusts (PCT) during the survey period. In one large PCT area, there were two SSSP. Eight SSSP operated across two PCT areas and two SSSP operated across three PCT areas.

No significant differences were observed between ‘respondent’ and ‘non-respondent’ SSSP in terms of area-level IMD score or self-reported quit rates (p = 0.35 and p = 0.11, respectively; Table 1). There were, however, significantly higher numbers of pregnant women reported to have set quit dates and to have successfully quit for at least four weeks by respondent SSSP, compared to non-respondent services (p < 0.01 and p < 0.01, respectively). Similarly, the number of CO validated quits (p = 0.003) was significantly higher in respondent compared to non-respondent services but there was no significant difference in CO validated quit rates (p = 0.92). Marginal statistical evidence was observed for SaToD being higher amongst respondents compared to non-respondents (p = 0.05; Table 1).

**Table 1:** Comparing routine data for ‘respondents’ and ‘non-repondents’ to the SSSP survey

**Variables**	**Completed survey (*n*** **= 121)**	**Did not complete survey (*n*** **= 20)**	**Comparison test**	**Test**
				**p-value**
Number set quit date	127 (80–211)	63.5 (48–92)	WMW	<0.01
[median (IQR)]				
Number SR four-week quitters	58 (34–82)	27.8 (16–45)	WMW	<0.01
[median (IQR)]				
Quit rate*	45.5 ± 11.5	43.0 ± 10.5	t-test	0.35
[Mean ± S.D.]				
SSSP area-level IMD score	22.7 ± 8.0	25.9 ± 8.3	t-test	0.11
[Mean ± S.D.]				
SaToD	14.7 ± 5.7	11.9 ± 6.6	t-test	0.05
[Mean ± S.D.]				

IQR, Interquartile Range; S.D., Standard Deviation; WMW, Wilcoxon-Mann–Whitney test; SSSP, Stop Smoking Service for Pregnant women; SR, Self-Reported; IMD, index of multiple deprivation; SaToD, smoking at time of delivery.

Of the 121 services that completed the survey, number set quit date and number four week quitters was available for 119 services and quit rate, IMD, and SaToD was available for 120 services.

Wilcoxon-Mann–Whitney Test used to assess potential differences in the underlying distribution of non-normally distributed variables for responding services compared to non-responding services. Two sample t-test used to assess potential differences in the mean of normally distributed variables for responding services compared to non-responding services.

*self-reported quit rate calculated as proportion of pregnant women who set a quit date that remain quit at four weeks.

### Service configuration

During April 2010 to March 2011, 67 (55%) of services provided at least some smoking cessation support to pregnant women through a ‘specialist pregnancy service’ (Table 2). This was defined as an organisation or organised system via which smoking cessation support was provided solely to pregnant women. However, these 67 services comprised 22 (33%) which supported pregnant smokers exclusively through specialist pregnancy services and 45 (67%) which provided supported via a combination of these and generic Stop Smoking Service (i.e. one which provides support to both pregnant and non-pregnant smokers). Twelve per cent of SSSP reported that smoking cessation support was provided to pregnant women on behalf of their service by midwives employed in a local hospital maternity unit. Two services reported that support was provided via a broad-ranging ‘Healthy Lifestyles’ service rather than a service solely dedicated to smoking cessation. 31% of services reported having a specific smoking in pregnancy budget for the 2010/11 financial year (Table 2). A total of 8 services (7%) were paid on a PbR basis (Table 2); of these five were located in the West Midlands of England.

**Table 2:** Organisation of English Stop Smoking Services for Pregnant women

		**Percentage**	***n***	Total*
**Service configuration**				
Support for pregnant women offered via:**	Generic Stop Smoking Service	57	69	121
	Specialist SSSP	55	67	
	Midwives employed by maternity unit	12	15	
	Healthy lifestyles’ service	2	2	
Funded by “payment by results”		7	8	121
Specific pregnancy budget		31	34	109
Have current specialist pregnancy advisor in post		76	92	121
Ever had a specialist pregnancy advisor in post		59	17	29†
Substantial changes have taken place at SSSP since March 2011		59	61	104
**Identification and referral**				
Identification of smokers:**	Self-reported smoking status only	39	46	118
	Exhaled CO only	27	32	
	Combination of exhaled CO and self-report	18	21	
	No policy exists/Not sure	9	11	
Referral of smokers:**	Opt-in	55	44	118
	Opt-out	37	65	
	No single policy/Not sure	8	9	
	Combined exhaled CO and opt-out	20	24	118
Most common referring group:	Community midwife	76	89	118
	Hospital midwife	17	20	
	Self-referral	3	4	
	Family doctor	2	2	
	Other	2	2	
	Clinician	1	1	
	Nurse	0	0	
	Health visitor	0	0	
	Children’s centre staff	0	0	
	Pregnancy outreach workers	0	0	
**Engaging with pregnant women**				
Staff member initially engaging with referred women:	Specialist stop smoking in pregnancy advisor	45	53	118
	Administrative staff	25	29	
	Specialist stop smoking in pregnancy manager/lead	13	15	
	Stop Smoking manager/advisor	12	14	
	Any staff member	3	3	
	Other	3	3	
	Midwife	1	1	
	Nurse	0	0	
	Pharmacist	0	0	
Mode of contact for engaging with referred women:	Telephone call	88	104	118
	Letter	6	7	
	Text message	3	3	
	Face-to-face in clinic	2	2	
	No standard contact method	2	2	
	Email	0	0	
	Home visit	0	0	
	Referred women establish contact	0	0	
Stage of pregnancy referrals most commonly received in:	First trimester	86	61	71††
	Second trimester	10	7	
	Third trimester	4	3	
**Service promotion**				
Setting in which smoking in pregnancy training provided:**	Mandatory training for maternity services midwives	58	68	118
	Optional training for maternity services midwives	58	68	
	Other	33	39	
	University training for student midwives	25	29	
	None	6	7	
Type of service promotion:**	Posters in antenatal clinics	93	100	108
	Training for midwives/Family doctor practice staff	91	98	
	Posters/flyers in community	82	89	
	Flyers etc. in maternity booking packs	70	76	
	Television/radio/local press	32	35	
	Obstetrician meetings	32	34	
	Other	30	32	
	Internet including social networking sites	19	20	
	None	2	2	

SSSP, Stop Smoking Service for Pregnant Women; CO, carbon monoxide; NRT, nicotine replacement therapy.

*total relates to number of SSSPs answering any part of that question. **question type allowed for multiple responses. †question was only asked of the 29 SSSP who said they did not have a specialist smoking in pregnancy advisor in post during 2010–11. ††this question was only asked of the 71 SSSP who said women were referred more commonly during a particular stage of their pregnancy.

Seventy-six percent of SSSP had a specialist pregnancy advisor in post, that is, an advisor who deals exclusively with pregnant women. Of those that did not, 41% had never had a staff member fulfilling that role (Table 2). The most frequent category for the duration that the specialist advisor had been in post was ‘more than three years’ (63%). The time during which those SSSP currently without a specialist advisor in post but had previously employed one (n = 17) ranged from ‘up to six months’ to ‘more than two years’.

### Identification and referral

Just under half (45%) of SSSP reported using exhaled CO to identify pregnant smokers as they attended antenatal health care. This comprised 27% that identified smokers solely through CO monitoring and 18% that used a combination of self-report and exhaled CO. A further 39% of SSSP reported using self-reports only (Table 2).

There are currently two models via which pregnant smokers are referred to a SSSP: opt-out referrals and opt-in referrals. For opt-out referrals, all identified smokers are referred unless the woman declines, whereas for opt-in referrals, smokers are asked if they would like to be referred. A total of 37% of SSSP reported that the policy at their maternity unit was to refer pregnant women identified as smokers via an opt-out referral pathway (Table 2) with the majority (55%) of services reporting referrals via an opt-in referral pathway, with the remainder being unsure. Although recommended by national guidance, just 20% of services reported using a combination of identifying pregnant smokers using exhaled CO and then referring those identified as smokers using an opt-out pathway (Table 2).

### Engaging with pregnant women

After receiving referrals, 88% of SSSP engaged with pregnant smokers by telephone with 58% using a specialist smoking in pregnancy advisor or manager to make first contact with referred women (Table 2). Administrative staff initially engaged with referred pregnant women in 25% of SSSP. A minority of SSSP also used letters (6%), text messages (3%) and face-to-face contact in the clinic (2%) to engage with pregnant smokers. Only 2% of SSSP reported no standard contact method although contact was made by specialist pregnancy advisors.

The majority (58%) of SSSP provided training on smoking in pregnancy during midwifery training at local hospitals (Table 2). Additionally, 25% of SSSP are involved with training of student midwives. Only 6% of SSSP did not report being involved with training midwives (Table 2). Almost all (88%) of SSSP reported promoting their pregnancy service. The most popular types of service promotion included training for midwives/family doctor practice staff (91%), posters in antenatal clinics (93%) and posters/flyers elsewhere in the community (82%; Table 2).

### Service delivery

The most common locations in which SSSP reported providing smoking cessation support to pregnant women were in the client’s own home (72%), children’s centres (72%) and in family doctor practices (71%; Table 3). The median number of locations used for delivering smoking cessation support (from a list of 12) was 5 (ranging from 1 to 9).

**Table 3:** Service delivery models employed by English Stop Smoking Services for Pregnant women

		**Percentage**	***n***	Total*
Location of smoking cessation support for pregnant women:**	Women's homes	73	80	110
	Children’s centres	73	80	
	Family doctor practices	72	79	
	Clinics	69	76	
	Health centres	63	69	
	Other community venues	59	65	
	Pharmacies	56	62	
	Other	10	11	
	Military	7	8	
	Mobile units	6	7	
	Prisons	5	5	
	Dentists	5	5	
Types of intervention provided:**	Behavioural support and dual NRT	86	95	110
	Behavioural support and single NRT	77	85	
	Relapse prevention	65	72	
	Behavioural support only	62	68	
	Financial incentive schemes	35	39	
	Dual NRT only (no behavioural support)	10	11	
	Single NRT only (no behavioural support)	9	10	
Most frequently used NRT products:**	Transdermal patches	93	79	85†
	Inhalator	87	74	
	Mini-lozenges	67	57	
	Gums	47	40	
	Mouth spray	13	11	
	Lozenges	12	10	
	Microtabs	11	9	
	Nasal spray	2	2	
Mode of NRT supply:**	Voucher to be redeemed at local pharmacy only	43	47	108†
	Combination of supply methods	28	31	
	Physician prescription only	20	22	
	Direct supply only	8	9	
Routine use of CO monitoring during smoking cessation treatment		89	98	110
Self-help materials provided to pregnant women		64	70	110
Types of self-help materials provided to pregnant women:**	Booklet/leaflet	97	68	70††
	Website	57	40	
	Generic text message advice	21	15	
	DVD	20	14	
	Other	9	6	
	Application on mobile phone/device	4	3	
	Generic email advice	3	2	
Agreement with statement ‘self-help materials are effective in helping women to stop smoking during pregnancy’:	Not at all	3	2	72††
	A little	29	21	
	Moderately	49	35	
	Very much	14	10	
	Extremely	6	4	
Agreement with statement ‘self-help materials should be included in routine care for smoking cessation during pregnancy’:	Not at all	0	0	70††
	A little	10	7	
	Moderately	29	20	
	Very much	39	27	
	Extremely	23	16	

SSSP, Stop Smoking Service for Pregnant Women; CO, carbon monoxide; NRT, nicotine replacement therapy.

*total relates to number of SSSPs answering any part of that question. **question type allowed for multiple responses. †these questions were only asked of the 110 SSSP who said they offered NRT to pregnant women. ††question was only asked of the subset of 72 SSSP who reported providing pregnant women with smoking cessation self-help materials.

On the basis that support may be tailored to each individual pregnant woman, we asked which combinations of behavioural support and nicotine replacement therapy (NRT) were offered during the year 2010–11 from the following: single therapy NRT only, dual therapy NRT only, behavioural support and single therapy NRT, behavioural support and dual therapy NRT, and behavioural support only; respondents could select more than one answer (Table 2). The majority (62%) of services reported providing psychosocial (behavioural) support without accompanying NRT to pregnant women. Behavioural support was also offered concomitantly with either single or dual therapy NRT i.e. a long lasting patch along with a short acting NRT product by 77% and 86% of services, respectively (Table 3). All respondent services reported providing some form of NRT to pregnant women and 9% and 10% provided either single or dual therapy NRT, respectively, to pregnant women without accompanying behavioural support. Many SSSP (65%) also reported providing ‘relapse prevention support’. We defined relapse prevention support as where women were supported following their cessation treatment programme to prevent relapse. Around a third (35%) of services provided smoking cessation support as part of a financial incentive scheme, defined as providing financial rewards to pregnant women upon reaching predefined smoking cessation targets (Table 3).

The most frequently used types of NRT products offered were transdermal patches (93%), inhalators (87%) and mini-lozenges (67%; Table 3). When asked how NRT was supplied to pregnant women, 43% of services did so using vouchers to be redeemed at local pharmacies, 20% reported NRT was provided by physician prescription, 8% directly supplied NRT to pregnant women whilst the remaining 28% reported using a combination of these methods (Table 3). The majority of services also said that exhaled CO testing was routinely used as part of smoking cessation treatment provided to pregnant women (89%; Table 3).

The majority (90%) of services reported providing intensive one-to-one behavioural support to pregnant women. Of these, 11% provided this solely in clinics, 26% provided this solely in women’s homes and the remaining 63% provided one-to-one support in both clinics and women’s homes. The median percentage of pregnant women accessing one-to-one support in the home was 54% compared to 50% for clinic-based support. Home visits took on average more time than clinic-based visits (mode time categories 30–60 minutes and 20–30 minutes, respectively; Table 4). The median number of different behavioural support types from the list of 10 (Table 4) selected by services was 4 (range 1–6).

**Table 4:** Proportion of pregnant women accessing behavioural support types across stop smoking services for pregnant women

	**Number SSSP providing support type**	**Median (IQR) percentage women accessing support type across all SSSP**	Mode time category per session in minutes ( *n *)*	Total**
Clinic based one-to-one support	97	50 (20–80)	20-30 (42)	108
Home based one-to-one support	81	54 (15–75)	30-60 (40)	
Telephone support	64	7 (3–15)	10-20 (11)	
Couple/family sessions	48	8 (4–14)	30-60 (15)	
Drop-in sessions	47	10 (4–20)	20-30 (10)	
Text message support	23	5 (2–10)	<5 (3)	
Open group sessions	16	5 (1–5)	10-20 (1); 20–30 (1)	
Other	8	7 (3–13)	10-20 (4)	
Closed group sessions	6	4 (2–5)	30-60 (1)	
Email support	2	3	.	

SSSP, Stop Smoking Services for Pregnant women; IQR, Interquartile Range.

*n relates to number of services that selected that time category option.

**total relates to number of services answering any part of question.

## Discussion

The study reported here is the first to describe how smoking cessation support is offered to pregnant women across England. Despite being relatively new, these services are well established in all parts of the country and offer a range of interventions from a variety of local community locations; the majority offer support to pregnant women from specialist pregnancy advisors and many smoking cessation services are specifically dedicated to pregnant women. England offers smoking cessation support to help any pregnant woman motivated to quit which is free at the point of use and our survey demonstrates the diversity of support currently offered. The services described provide a potentially useful model for other countries.

Smokers in the UK are estimated to be four times more likely to quit if they use a Stop Smoking Service [22], however, around only 15% of pregnant women who smoke access these services in England [20]. We found that only one third of services employed ‘opt-out’ referral pathways. These involve antenatal services passing on the details of any pregnant woman who smokes to Stop Smoking Services and, although such referral pathways are recommended in national guidance,[16] further evidence for their effectiveness is needed. One recent UK study found identifying pregnant smokers using carbon monoxide testing and referring to SSSP via an opt-out pathway increased referrals, but not successful four week CO-validated quits [23]. Additional methods to maximise uptake of services include providing training at mandatory midwifery events to keep smoking cessation on their agenda [12,24,25] and providing promotional materials such as posters and flyers in both hospital and community locations. Although diverse methods for engaging pregnant smokers in support from SSSP appear to be widely used, the relative effectiveness of these has yet to be determined.

Self-reported smoking status is documented as potentially being unreliable in the general population where non-smoking tends to be over reported [26,27] and, due to the added social stigma of smoking in pregnancy, this may also be problematic for obtaining smoking status at this time too [28,29]. Although exhaled breath carbon monoxide testing is a practical method for validating smoking status [29], less than half of SSSP use exhaled CO to identify pregnant women which suggests the need for this practice to be recognised more widely by both service managers and at NHS Acute Trusts responsible for maternity care.

Foetal safety remains a concern for the use of NRT during pregnancy, although the consensus is that harm is reduced compared to maternal smoking [30,31]. Consistent with findings from a qualitative study of three UK Stop Smoking Services [25], our survey showed that all responding services in England offer NRT to pregnant women as part of their smoking cessation treatment. However, there is currently insufficient evidence to conclude whether NRT in pregnancy is either effective or safe [3,32,33], as demonstrated in a recent meta-analysis of six randomised controlled trials of NRT use for smoking cessation in pregnancy [34]. We found that transdermal patches were the most frequently provided form of NRT, despite previous work showing patch compliance to be low in pregnant women [32,35].

Most of the literature evaluating NRT use in pregnancy relates to single therapy NRT [34,36] yet the majority (86%) of services have reported offering higher-dose, dual therapy NRT which involves using both a transdermal patch and a short acting NRT formulation concurrently. Further research is needed to evaluate the utility of this practice. One recent correlation study found dual therapy NRT to be associated with smoking cessation in women attending English SSSP [37], although this has yet to be assessed in a randomised control trial. Around 1 in 10 services also offer NRT products to pregnant women in the absence of behavioural support. However, there is strong evidence that intensive behavioural support is effective for smoking cessation during pregnancy [3], and the logic of providing an unproven treatment such as NRT without this is questionable.

Although offering a flexible client-centred service is generally considered to be key to smoking cessation support [16,38], there is little evidence underpinning how this should be done. Most services seem to attempt this by using a range of methods to engage with pregnant women who have been referred to the service, providing smoking cessation support in a number of community locations and offering a range of behavioural support types. Almost 90% of services offered intensive one-to-one support either in the client’s home or clinic setting and home visits were reported, on average, to take more time than clinic-based ones resulting, in higher service delivery costs for these. However, there is no evidence that home –delivered support is more effective; Brose *et al*. reported no significant difference in CO validated four week abstinence for pregnant women receiving home visits compared to attending support in specialist clinics in England [37].

The extent to which financial incentives for smoking cessation in pregnancy are now being used in England, is an interesting. A meta-analysis of pooled results by Lumley *et al*. from four trials showed financial incentives for smoking cessation during pregnancy were significantly more effective than other intervention strategies [3]. All of these trials took place in the USA and UK national guidance has called for research to establish whether these types of interventions could be effective if used in the UK NHS [16]. What appears to have happened in practice is that a proportion of stop smoking services have added incentives to their existing service provision despite limited evidence. Just over a third of services were using these types of interventions, with many located in one English region that was piloting their delivery over a two year period. This raises important implications for future research as evidence of their effectiveness in routine care could inform future service developments both in the UK and elsewhere.

This study had a number of strengths as well as limitations. We received a high response rate of 86%, suggesting that the results are likely to be representative of SSSP in England. In addition, responding and non-responding services did not differ in terms of quit rates or deprivation score, offering further reassurance about the representativeness of findings. Responding services were significantly ‘larger’, i.e. more pregnant women attended and set quit dates and stopped smoking using these services, compared with non-responding ones. This may reflect that larger services were better able to find a member of staff with the time to complete the survey. To the extent that these differences may have influenced the results of the survey, the comprehensiveness of models of service delivery may have been overestimated. A potential limitation of the study is that data are self-reported and as such may be unreliable. We do not anticipate, however, that service managers would have been motivated to provide incorrect information. Inaccuracies may have arisen through misinterpretation of the online survey, however, we addressed this by providing ‘pop-up’ definitions where, appropriate. Additionally, at the outset, we recognised potential difficulties in summarising complex service models in survey questions and categories and attempted, address this with thorough piloting.

## Conclusions

This work illustrates that the NHS SSSP provide flexible client-centred services to help pregnant women stop smoking in England, and identifies a number of elements of service provision that merit further research and evaluation.

## Abbreviations

SSSP: Stop smoking services for pregnant women; PbR: Payment by results; IMD: Index of multiple deprivation; SaToD: Smoking at time of delivery; CO: Carbon monoxide; NRT: Nicotine replacement therapy; PCT: Primary care trust.

## Competing interests

The authors declare that they have no competing interests.

## Authors’ contributions

SF developed the survey questions, designed the online survey, acquired the data and drafted and revised this manuscript. SC helped conceive the study, assisted in developing the survey, assisted with day-to-day troubleshooting during the data collection phase and contributed to the preparation of this manuscript. TC conceived the study and made substantial contributions to both the survey development and the preparation of this manuscript. FN helped conceive the study, provided invaluable input to a section of the survey (smoking cessation self-help materials) specific to his area of expertise and contributed to the survey development and preparation of this manuscript. LB conceived the design of the study, drafted the preliminary survey questions, contributed to the survey development and made substantial contributions to the drafting and preparation of this manuscript. All authors read and approved the final manuscript.

## Pre-publication history

The pre-publication history for this paper can be accessed here:

http://www.biomedcentral.com/1472-6963/14/107/prepub
